# Photolithographic shape control of DNA hydrogels by photo-activated self-assembly of DNA nanostructures

**DOI:** 10.1063/1.5132929

**Published:** 2020-03-20

**Authors:** Yu Kasahara, Yusuke Sato, Marcos K. Masukawa, Yukiko Okuda, Masahiro Takinoue

**Affiliations:** Department of Computer Science, Tokyo Institute of Technology, Yokohama, Kanagawa 226-8502, Japan

## Abstract

We report a photolithographic method for the shape control of DNA hydrogels based on photo-activated self-assembly of Y-shaped DNA nanostructures (Y-motifs). To date, various methods to control the shape of DNA hydrogels have been developed to enhance the functions of the DNA hydrogel system. However, photolithographic production of shape-controlled DNA hydrogels formed through the self-assembly of DNA nanostructures without the use of radical polymerizations has never been demonstrated, although such a method is expected to be applied for the shape-control of DNA hydrogels encapsulating sensitive biomolecules, such as proteins. In this study, we used a photo-activated linker to initiate the self-assembly of Y-motifs, where the cross-linker DNA was at first inactive but was activated after UV light irradiation, resulting in the formation of shape-controlled DNA hydrogels only at the UV-exposed area produced by photomasks. We believe that this method will be applied for the construction of biohybrid machines, such as molecular robots and artificial cells that contain intelligent biomolecular devices, such as molecular sensors and computers.

## I. INTRODUCTION

DNA hydrogels^1–5^ have attracted much attention^6–9^ due to their programmable functions, biocompatibility, and their applicability, such as in drug delivery systems,^10,11^ cell-free protein synthesis,^12^ artificial cytoskeletons for artificial cells,^13^ mechanical micromachines,^14^ and locomotive micromaterials.^15^ DNA hydrogels are classified into three types as follows: (i) “DNA-motif hydrogels,” which mainly consist of DNA and are formed through the self-assembly of Y-/X-/T-branched DNA nanostructures;^1,3,16,17^ (ii) “DNA-entanglement hydrogels,” which mainly consist of DNA and are formed through the entanglement of long polymerized DNA;^15,18^ (iii) “DNA-modified polymer hydrogels,” which mainly consist of non-DNA polymers and are formed through the cross-linking by DNA strands modified to the polymer, such as DNA-cross-linked polyacrylamide hydrogels^4,5,14^ and DNA-cross-linked polypeptide hydrogels.^19^ In all the cases, (i)–(iii), the gelation of the DNA hydrogels is based on specific DNA hybridization reactions between complementary DNA strands. Thus, by designing DNA sequences, DNA hydrogels can acquire various stimuli-responsive properties, such as pH-triggered gelation,^20^ pH- and ligand-induced drug release,^12^ thermo-triggered gelation,^21,22^ photo-induced gelation,^22,23^ ion-stimulated gelation,^24^ sensitivity to heavy metal ions,^25^ gelation triggered by a short single-stranded DNA,^16^ shape memory,^18,26^ and mechanical motion.^14^

To enhance the functions of DNA hydrogels, various approaches have been used to control their shape. The most frequently used method for shape control is the gelation of DNA hydrogel using a designed mold.^15,18,26^ Recently, researchers have achieved the shape control of DNA hydrogels without molds. First, Li *et al.* demonstrated three-dimensional bioprinting of millimeter-sized DNA-cross-linked polypeptide hydrogels, based on the ink-jet-like discharge from the scanning nozzle.^19^ Wang *et al.* demonstrated the surface-initiated pattern formation of DNA-motif hydrogels, based on the clamped hybridization chain reaction,^16^ where DNA strands were elongated from the initiator DNAs micropatterned on a glass surface, resulting in DNA-motif hydrogels patterned in the scale of several hundred micrometers. In addition, shape-controlled DNA-motif hydrogel formation based on patterned photo-irradiation was reported. For example, Shimomura *et al.* reported photothermal fabrication of DNA-motif hydrogels, using computer-generated laser holograms,^22^ where capping DNAs were removed by laser heating and Y-shaped DNAs self-assembled at the patterned laser irradiation points. Cangialosi *et al.* demonstrated the photolithographic formation of DNA-cross-linked polyacrylamide hydrogels, with arbitrary shapes,^14^ where radical polymerization produced DNA-cross-linked polyacrylamide hydrogels by ultra-violet (UV) light irradiation combined with a photo-initiator, acrylamide monomers, and acrydite-modified DNA cross-linkers. These methods based on patterned photo-irradiation are highly scalable because of their flexible, parallel, and rapid generation features. However, the free radical reaction often damages sensitive biomolecules such as proteins. Therefore, photolithographic formation of shape-controlled DNA-motif hydrogels based only on the photoinitiated self-assembly of branched DNA nanostructures, without the use of radical polymerization, should be explored for the functionalization with proteins or the *in situ* biological experiments. The photoinitiated physical cross-linkers will allow us to encapsulate sensitive biomolecules in shape-controlled DNA-motif hydrogels and will be useful for the construction of biohybrid machines, such as molecular robots^27^ and artificial cells.^28^

**FIG. 1.**
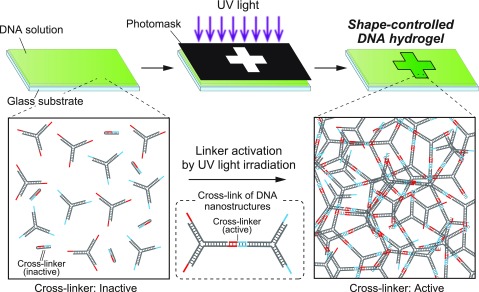
Conceptual illustration of the photolithographic formation of shape-controlled DNA-motif hydrogels based on the photo-activated self-assembly of DNA nanostructures.

In this paper, we propose a photolithographic method for the shape control of DNA-motif hydrogels by photo-activated self-assembly of DNA nanostructures. Figure 1 shows the conceptual illustration of the photolithographic formation of shape-controlled DNA-motif hydrogel using UV light irradiation. The cross-linker DNA is first inactivated by forming a hairpin shape, but the cross-linker DNA is activated after UV light irradiation because the covering DNA sequence that forms the hairpin shape has photo-cleavable modifications and is removed after the UV light irradiation. This mechanism allows us to form DNA-motif hydrogels only at the UV-exposed area in the DNA solution. By UV light irradiation through a photomask, we can construct a microstructure of DNA-motif hydrogels with an arbitrary shape.

## II. RESULTS AND DISCUSSION

### A. Strategy of DNA-motif hydrogel formation by UV light irradiation and DNA sequence design

**FIG. 2.**
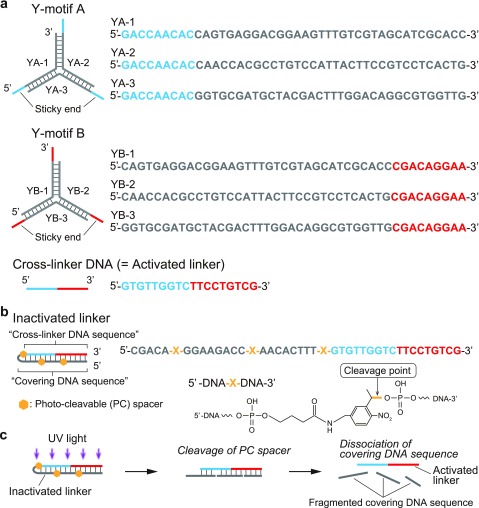
Designs of DNAs. (a) Design and sequences of Y-shaped DNA nanostructures (named “Y-motif A” and “Y-motif B”) and cross-linker DNA sequence. The cross-linker DNA sequence has sequences complementary to the sticky ends of Y-motif A and Y-motif B. (b) Design and sequence of inactivated linker. The inactivated linker has the cross-linker DNA sequence (cyan and red) and the covering sequence (gray) with photo-cleavable (PC) spacers at three “X” positions. (c) The mechanism of activation of the inactivated linker. The PC spacers are cleaved by the irradiation of UV light with a wavelength of 300–350 nm, causing the covering DNA sequences to dissociate, releasing the activated linker.

For the photolithographic formation of shape-controlled DNA-motif hydrogels, we designed two types of Y-shaped DNA nanostructures (named “Y-motif A” and “Y-motif B”) and a single-stranded “cross-linker DNA” [Fig. 2(a)]. Y-motifs A and B are three-branched nanostructures composed of 16-base double-stranded stems and 9-base single-stranded sticky ends, which are constructed from three single-stranded DNAs (ssDNAs) (YA-1, YA-2, and YA-3 for Y-motif A; YB-1, YB-2, and YB-3 for Y-motif B). The cross-linker DNA has sequences that hybridize with the sticky ends of Y-motifs A and B. Therefore, the sticky ends of Y-motifs A and B can be connected by the cross-linker DNA and then form a DNA-motif hydrogel with a network structure.

To realize the photo-activated gelation of Y-motifs A and B using UV light irradiation, we designed a hairpin-shaped DNA, named inactivated linker [Fig. 2(b)] that is the cross-linker DNA covered with a photo-cleavable sequence, so that Y-motifs A and B do not cross-link before UV light irradiation. The inactivated linker has three photo-cleavable spacers (PC spacers), which are marked as “X” in Fig. 2(b). Upon irradiation of UV light with the wavelength range of 300–350 nm, the covering DNA sequence is split into three shorter sequences at the PC spacers (8 bases, 8 bases, and 5 bases) and the shortened covering DNA sequence easily dissociates from the cross-linker DNA sequence [Fig. 2(c)]. Subsequently, the inactivated linker returns to the intact cross-linker DNA with cross-linking activity; hereafter, the intact cross-linker DNA will be called “activated linker.” This design ensures that a mixed solution of Y-motifs A and B and inactivated linker does not gelate until exposed to UV light.

In order to achieve the gelation of Y-motifs A and B at room temperature (approximately 25 °C, in this study), we designed DNA sequences, as shown in Figs. 2(a) and 2(b), using Nucleic Acid Package (NUPACK).^29^ First, we confirmed that the melting temperature of the stems of Y-motifs A and B is 65 °C; that of the sticky end of Y-motif A is 35 °C; and that of Y-motif B is 37 °C (Fig. S1 in the supplementary material), where the melting temperature is defined as the temperature at which the rates of the hybridized and dissociated states are both 50%. The calculation results indicate that Y-motifs A and B and activated linker can gelate at 25 °C. Next, we numerically confirmed the melting temperature of the hairpin structure of the inactivated linker to be 81 °C (Fig. S2). After UV light irradiation, the covering DNA sequence of the inactivated linker is cleaved into three shorter DNA strands, whose melting temperatures are numerically calculated as <0 °C, 29 °C, and 1 °C, respectively, which are lower than 81 °C; thus, the resultant three shorter DNA strands are easy to dissociate [Fig. 2(c)]. Therefore, the DNA solution irradiated with UV light transitions to DNA-motif hydrogel at 25 °C. All calculations were performed on the condition that each sticky end concentration was 7.5 *μ*M (=2.5 *μ*M × 3, i.e., 3 times the concentration of Y-motif) and NaCl concentration was 100 mM.

### B. Electrophoretic verification of Y-motif formation

**FIG. 3.**
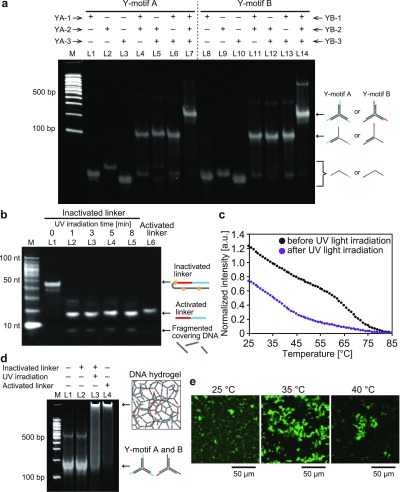
(a) Confirmation of Y-motif DNA formation using non-denaturing PAGE. M: 100-bp DNA ladder marker; L1–L3: YA-1, YA-2, or YA-3, respectively (2.5 *μ*M each); L4: YA-1 and YA-2 (2.5 *μ*M each); L5: YA-2 and YA-3 (2.5 *μ*M each); L6: YA-1 and YA-3 (2.5 *μ*M each); L7: YA-1, YA-2, and YA-3 (2.5 *μ*M each); L8–L10: YB-1, YB-2, or YB-3, respectively (2.5 *μ*M each); L11: YB-1 and YB-2 (2.5 *μ*M each); L12: YB-2 and YB-3 (2.5 *μ*M each); L13: YB-1 and YB-3 (2.5 *μ*M each); and L14: YB-1, YB-2, and YB-3 (2.5 *μ*M each). (b) Confirmation of activation of inactivated linker (i.e., photo-cleavage and dissociation of the covering DNA sequence from the inactivated linker) using denaturing PAGE. M: 10-bp DNA ladder marker; L1–L5: 1 *μ*M inactivated linker with UV light irradiation for 0 min, 1 min, 3 min, 5 min, and 8 min, respectively; L6: 1 *μ*M activated linker without UV light irradiation. (c) Melting analysis of the inactivated linker before and after UV light irradiation. (d) Confirmation of DNA-motif hydrogel formation by UV light irradiation using non-denaturing PAGE. M: 100-bp DNA ladder marker; L1: 2.5 *μ*M Y-motif A and 2.5 *μ*M Y-motif B, without UV light irradiation; L2: 2.5 *μ*M Y-motif A, 2.5 *μ*M Y-motif B, and 7.5 *μ*M inactivated linker, without UV light irradiation; L3: 2.5 *μ*M Y-motif A, 2.5 *μ*M Y-motif B, and 7.5 *μ*M inactivated linker, with UV light irradiation; L4: 2.5 *μ*M Y-motif A, 2.5 *μ*M Y-motif B, and 7.5 *μ*M activated linker, without UV light irradiation. (e) Microscopy image of aggregated DNA-motif hydrogel microparticles formed by UV light irradiation. The temperatures when UV light was irradiated were 25 °C, 35 °C, and 45 °C, respectively.

First, the formation of Y-motif A and Y-motif B was examined with non-denaturing polyacrylamide gel electrophoresis (PAGE) [Fig. 3(a)]. The DNA solution used for non-denaturing PAGE contained 2.5 *μ*M of each ssDNA (YA-1, YA-2, and YA-3 for Y-motif A; YB-1, YB-2, and YB-3 for Y-motif B), 100 mM NaCl, 20 mM Tris-HCl (pH 8.0), and 1×SYBR Gold (DNA stain). The observed bands in lanes L1–L3 and L8–L10 in Fig. 3(a) indicate each ssDNA. Second, for the combination of two of the three ssDNAs, the bands shifted up (lanes L4–L6 and L11–L13). Finally, when all the three ssDNAs were contained, the bands shifted up further (lanes L7 and L14), which correspond to Y-motifs A and B. Based on the above results, we conclude that Y-motifs A and B were formed as designed in this buffer condition and at 25 °C.

### C. Cleavage of inactivated linker by UV light irradiation

We confirmed the cleavage reaction of the inactivated linker by UV light irradiation, using denaturing PAGE [Fig. 3(b)]. A DNA solution consisting of 1 *μ*M inactivated linker (or 1 *μ*M activated linker), 100 mM NaCl, 20 mM Tris-HCl (pH 8.0), and 1×SYBR Gold was prepared. After UV light was irradiated on the solution for 0–8 min with a high-pressure mercury lamp, we evaluated the effect of the duration of the UV light irradiation on PC spacer cleavage rate, using denaturing PAGE [Fig. 3(b)]. Before irradiating with UV light (0 min, lane L1), the band of inactivated linker was observed at the highest position; after irradiation (1–8 min, lane L2–L5), the brightest band shifted below and a much shorter band appeared under the brightest band, indicating that the inactivated linker was cleaved into shorter DNA strands. The bands of the cleaved inactivated linker (lane L2–L5) were compared with the activated linker, which is used as control (lane L6). The brightest band was the generated activated linker and the lower band was the fragmented covering sequence, which suggests that UV light irradiation cleaved the inactivated linker at the designated positions of the PC spacer. In addition, since the conditions of 3–8 min of UV light irradiation (lane L3–L5) were similar, a 3-min irradiation was considered to be enough for the cleavage of the inactivated linker.

Next, we confirmed the cleavage reaction based on melting analysis [Fig. 3(c)]. The melting curves for the inactivated linker, before and after UV light irradiation, were observed using the fluorescence intensity detection mode of a real-time PCR system (CFX Connect, Bio-Rad). The DNA solution contained 5 *μ*M inactivated linker, 100 mM NaCl, 20 mM Tris-HCl (pH 8.0), and 1×SYBR Green I (DNA stain). UV light was irradiated for 3 min with the high-pressure mercury lamp, only on the sample marked “after UV light irradiation” in Fig. 3(c). The fluorescence intensity of the melting curve of the inactivated linker after UV light irradiation was lower than that of the inactivated linker before UV light irradiation, indicating that the covering sequences were spontaneously dissociated from the activated linker sequence after UV light irradiation, as designed.

### D. Verification of the formation of DNA-motif hydrogel using the inactivated linker and UV light irradiation

We confirmed the formation of the DNA-motif hydrogel using the inactivated linker and UV light irradiation by non-denaturing PAGE [Fig. 3(d)]. Here, we compared four conditions; Y-motifs A and B (lane L1), those with inactivated linker but without UV irradiation (lane L2), those with inactivated linker and UV irradiation (lane L3), and those with activated linker (lane L4). A DNA solution consisting of 2.5 *μ*M Y-motif A, 2.5 *μ*M Y-motif B, 7.5 *μ*M inactivated linker (or 7.5 *μ*M activated linker, or no linkers), 100 mM NaCl, 20 mM Tris-HCl (pH 8.0), and 1×SYBR Gold was prepared. UV light was irradiated for 3 min with the high-pressure mercury lamp. Lane L2 showed the same electrophoretic profile as lane L1, indicating that the inactivated linker did not connect Y-motifs A and B, when the sample was not irradiated with UV light. Lane 3 showed the same electrophoretic profile as lane L4, that is, almost all DNA was trapped at the top of the well of the polyacrylamide gel because of DNA aggregation (network formation) and did not move during electrophoresis. Thus, these results demonstrate that the Y-motifs A and B and the inactivated linker did not form a DNA-motif hydrogel without UV light irradiation but specifically formed it after UV light irradiation.

Figure 3(e) shows the confocal laser scanning microscope images of the DNA-motif hydrogel of the Y-motifs A and B, formed with the inactivated linker after UV light irradiation. We irradiated UV light on a DNA solution that contained 2.5 *μ*M Y-motif A, 2.5 *μ*M Y-motif B, and 7.5 *μ*M of inactivated linker, dissolved in 20 mM Tris-HCl (pH 8.0) buffer, 100 mM NaCl, and 1×SYBR Gold using the high-pressure mercury lamp for 3 min at 25 °C, 35 °C, and 40 °C. Under all three conditions, we observed DNA-motif hydrogels, several micrometers in diameter [Fig. 3(e)], and DNA-motif hydrogel microparticles were larger under the 35 °C condition.

### E. Photolithographic formation of shape-controlled DNA-motif hydrogels

**FIG. 4.**
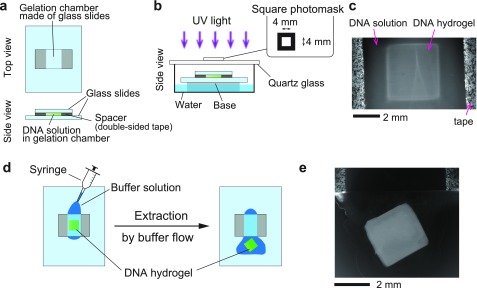
DNA-motif hydrogel formation with UV light irradiation. (a) The schematic illustration of the gelation chamber made of two cover glasses. (b) The schematic illustration of UV irradiation through the photomask. To prevent evaporation of the DNA solution, a water bath was placed under the gelation chamber in a closed vessel. (c) The photograph of a prepared DNA-motif hydrogel inside the gelation chamber. (d) The schematic illustration of how to extract the prepared DNA-motif hydrogel from the gelation chamber. (e) The photograph of an extracted DNA-motif hydrogel.

Figures 4(a) and 4(b) show the experimental setup for photo-activated generation of shape-controlled DNA-motif hydrogels using UV light irradiation. A DNA solution containing 25 *μ*M Y-motif A, 25 *μ*M Y-motif B, and 75 *μ*M inactivated linker, in 200 mM NaCl and 20 mM Tris-HCl (pH 8.0) buffer, was introduced into the glass-slide chamber [Fig. 4(a)]. UV light was irradiated on the chamber for 10 min, using a xenon lamp with a 313 nm bandpass filter, through a square photomask [Fig. 4(b)]. The photomask was fabricated using standard photolithographic methods. Figure 4(c) shows the fluorescence microscopy image of the 4 mm × 4 mm square-shaped DNA-motif hydrogel generated by UV light irradiation. After the formation of the shape-controlled DNA-motif hydrogel, we extracted the square DNA-motif hydrogel from the glass-slide chamber by passing the buffer (20 mM Tris-HCl (pH 8.0) and 200 mM NaCl) using a syringe (2.5 ml, TERUMO) and a needle (27 G (S · B)×19 mm (3/4″), TERUMO) [Fig. 4(d)]. Figure 4(e) shows the fluorescence microscopy image of the extracted square-shaped DNA-motif hydrogel. This result demonstrates that the structure of the DNA-motif hydrogel could maintain its shape without the glass-slide chamber.

**FIG. 5.**
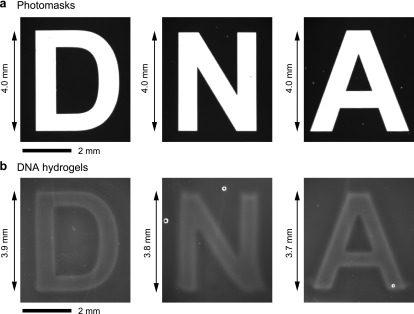
The formation of shape-controlled DNA-motif hydrogel with UV light irradiation. (a) The photograph of photomasks for the characters, “D,” “N,” and “A.” (b) The photographs of prepared shape-controlled DNA-motif hydrogels with the shapes of “D,” “N,” and “A.”

We demonstrated the formation of DNA-motif hydrogel with the shapes “D,” “N,” and “A” (Fig. 5), where the experimental conditions were the same as that for experiments in Fig. 4. Figure 5(a) shows bright-field microscopy images of the photomasks for “D,” “N,” and “A.” The microscopy images of the constructed DNA-motif hydrogel with the shapes of “D,” “N,” and “A” are shown in Fig. 5(b). Although the size of the constructed DNA-motif hydrogel was 0.1–0.3 mm smaller than that of the photomasks, shape-controlled DNA-motif hydrogels were successfully generated.

### F. Investigation of the resolution of the DNA-motif hydrogels formed using the photolithographic method

**FIG. 6.**
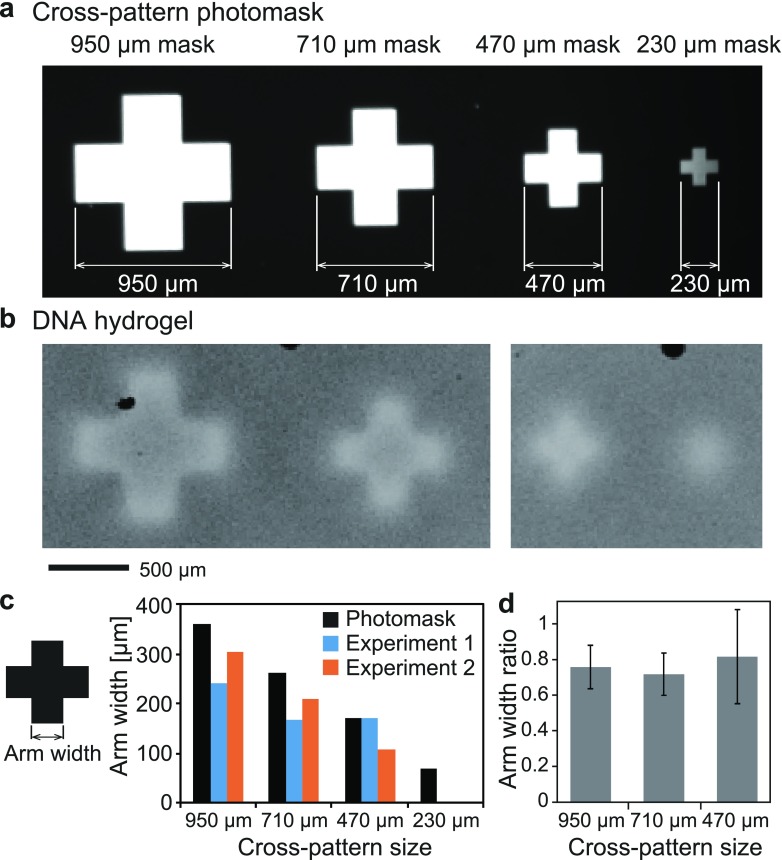
Investigation of the resolution of this method. (a) The photograph of photomasks for cross patterns with different sizes. (b) The photographs of the prepared shape-controlled DNA-motif hydrogels. (c) Comparison of arm width of prepared DNA-motif hydrogels with that of photomasks. (d) The arm width ratio of prepared DNA-motif hydrogels to that of photomasks.

In order to investigate the resolution of the photolithographic method used for the formation of shape-controlled DNA-motif hydrogels, we measured the size difference between cross-patterned photomasks and DNA-motif hydrogels generated using the photomasks [Fig. 6(a)], where the experimental conditions were the same as the experiments in Fig.4. Figure 6(a) shows the bright-field microscopy images of the cross-patterned photomasks with different sizes (950, 710, 470, and 230 *μ*m in width). Figure 6(b) shows the fluorescence microscopy images of DNA-motif hydrogels generated using these photomasks. When the photomasks with 950- and 710-*μ*m width were used, the shape of the DNA microgel was well reproduced. However, the outline of the formed DNA-motif hydrogel was a little blurred when the photomask with 470-*μ*m width was used, and it could not be identified when the photomask with 230-*μ*m width was used. Since a small amount of activated linker produced by UV light irradiation diffused to the non-irradiated region during gelation, the microstructure with 470-*μ*m width was blurred and did not gelate well in the case of the 230-*μ*m wide microstructures. Further, to quantify the resolution of this method, we compared the arm width of the cross pattern of the photomask and that of the formed DNA-motif hydrogels [Fig. 6(c)]. The DNA-motif hydrogel microstructures were ∼25% smaller than the photomask patterns [Fig. 6(d)], which was probably due to the shrinkage of the hydrogel caused by the cross-linking between monomers (here, Y-motifs). These results suggest that the resolution of this method was 100–200 *μ*m under these experimental conditions. To improve the resolution, the further optimization of parameters such as the distance between masks and the DNA solution layer, the size/type of the light source, and the power of irradiation should be performed.

## III. CONCLUSION

In this study, we designed a photo-activated gelation system for the photolithographic shape-control of DNA-motif hydrogel (Fig. 1). We confirmed that the designed Y-motifs and photo-activated linker (Fig. 2) worked to generate the DNA-motif hydrogel in a sequence-specific manner, based on the PAGE analyses and the microscopy observations (Fig. 3). The DNA-motif hydrogel was formed by a patterned irradiation of UV light and maintained its shape after extraction from the irradiation chamber (Fig. 4). Moreover, we demonstrated the formation of shape-controlled DNA-motif hydrogels using a photolithographic technique (Fig. 5). Finally, we estimated the resolution of this method (Fig. 6). The concept of the photo-activated gelation system of DNA-motif hydrogel was successfully demonstrated.

From this study, we found that the shrinkage of the hydrogel probably affected the size of the generated DNA-motif hydrogel, which was approximately 25% smaller than the photomask patterns; therefore, the size of the photomask must be designed ∼25% larger than desired size of the DNA-motif hydrogel. In addition, the diffusion of activated linkers during gelation may have affected the resolution of the DNA-motif hydrogel microstructures formed. The increase in the viscosity of the solution would improve the resolution by decreasing diffusion; moreover, the increase in the concentration of Y-motifs and inactivated linker could improve the resolution due to the acceleration of the gelation rate. The use of high-precision light source with a collimator also will improve the resolution.

Although we demonstrated only one type of DNA design for Y-motifs and inactivated linker, various types of Y-motifs and linkers can be designed to extend the functions of the DNA-motif hydrogel system. For example, complex-shaped hydrogels such as multilayer gels can be created by stacking DNA-motif hydrogels with different set of Y-motif and linker sequences based on the multi-step layer-by-layer photolithography. The phase-separation of microstructures of DNA-motif hydrogels^30^ is also available to construct complex-shaped DNA-motif hydrogels. In addition, DNA-motif hydrogels have biocompatibility due to enzymatical degradation (Fig. S3) and will be applied to *in vivo* application. Thus, photocleavage-based gelation could be versatile for many applications based on DNA-motif hydrogels. In the future, a shape-controlled and photo-responsive DNA-motif hydrogel can promote the construction of functional bodies or molecular computing devices for intelligent drug delivery systems, molecular robots, artificial cells, and biohybrid machines.

## IV. METHODS

### A. Materials

The DNAs YA-1, YA-2, YA-3, YB-1, YB-2, and YB-3 [Fig. 1(a)] were purchased as custom synthesized oligo DNAs and purified by oligonucleotide purification cartridge (OPC) from Eurofins Genomics, Japan. A fluorescent probe sequence named Y-2_FAM (5′-[FAM]-CAACCACGCCTGTCCATTACTTCCGTCCTCACTG-3′) was also purchased as a custom synthesized oligo DNA and purified by OPC from Eurofins Genomics. Y-2_FAM had the same sequence as the stem part of YA-2 and YB-2 and was modified with 6-carboxyfluorescein group (FAM) (maximum absorbance wavelength: 492 nm; maximum emission wavelength: 517 nm) at the 5′-terminal; therefore, Y-motifs A and B can be labeled with FAM by partially using Y-2_FAM together with YA-2 or YB-2. The inactivated and activated linker DNAs were purchased as custom synthesized oligo DNAs purified by high performance liquid chromatography (HPLC) from Tsukuba Oligo Service Co., Ltd. Sodium chloride (NaCl), bromophenol blue (BPB), glycerol, urea, ammonium peroxodisulfate (APS), *N,N,N′,N′*-tetramethylethylenediamine (TEMED), bovine serum albumin (BSA), acetone, isopropyl alcohol (IPA), and 1,1,1,3,3,3-hexamethyldisilazane (HMDS) were purchased from Fuji Film Wako Pure Chemical Industries, Ltd. 1 M Tris-HCl (pH 8.0) solution and 10 000×SYBR Gold solution were purchased from Life Technologies Japan. 10 000×SYBR Green I solution was purchased from Takara Bio. 0.5 M ethylenediaminetetraacetic acid (EDTA) (pH8.0) solution was purchased from Nippon Gene. 40% (w/v) acrylamide/bis solution (29:1) and 5×Tris-borate-EDTA (TBE) buffer were purchased from Nacalai Tesque. The 100-bp DNA ladder marker (Quick-Load 100 bp DNA Ladder) and the 10-bp DNA ladder maker (10 bp DNA Step Ladder) were purchased from New England Biolabs Japan and Promega Corporation, respectively. A photoresist S1818 (MICROPOSIT S1818G Photoresist) and a developer NMD-3 (OFPR-NMD-3) were purchased from Shipley and Tokyo Ohka Kogyo Co., Ltd., respectively. The chromium (1–5 mm, 99.9%) and gold (gold wire, φ0.25 mm, 99.95%) for deposition were purchased from Niraco Co., Ltd. The ultra-pure water was generated using Direct-Q UV (Merck Millipore).

### B. Polyacrylamide gel electrophoresis (PAGE)

The non-denaturing 8% (w/w) polyacrylamide gels were prepared by mixing 2 ml of 40% (w/v) acrylamide/bis solution and 2 ml of 5×TBE buffer with ultra-pure water (total volume was 10 ml), followed by the addition of 150 *μ*l of APS and 3 *μ*l of TEMED for gelation. The denaturing 10% (w/w) polyacrylamide gels containing 8 M urea were prepared by mixing 2.5 ml of 40% (w/v) acrylamide/bis solution, 2 ml of 5×TBE buffer, and 4.8 g of urea with ultra-pure water (total volume was 10 ml), followed by the addition of 150 *μ*l of APS and 3 *μ*l of TEMED for gelation. PAGE and pre-running were performed with 1×TBE buffer under a constant voltage of 150 V for the non-denaturing PAGE and 200 V for the denaturing PAGE, using an electrophoresis power supply (BP-T5, BIO-CRAFT). We made the loading sample solution by mixing the sample solution with an equal volume of 2×non-denaturing/denaturing loading buffer, and then we applied the loading sample solutions onto the wells of the polyacrylamide gel, after 30 min pre-running. Non-denaturing/denaturing PAGE was performed for 30 min. After electrophoresis, the polyacrylamide gel was stained with 1×TBE buffer containing 1×SYBR Gold for approximately 10 min. The gel was observed on an imaging device (Gel doc EZ, BIO-RAD). The 2×non-denaturing loading buffer contained 0.05% (w/w) BPB, 10% (w/w) glycerol, and 50 mM EDTA; the 2×denaturing loading buffer contained 0.05% (w/w) BPB, 10% (w/w) glycerol, 50 mM EDTA, and 8M urea.

### C. Confirmation of Y-motif formation by PAGE

We confirmed the formation of Y-motifs A and B by PAGE [Fig. 3(a)]. In Fig. 3(a), each DNA solution contained 2.5 *μ*M of each ssDNA, 100 mM NaCl, 20 mM Tris-HCl (pH 8.0), and 1×SYBR Gold. The DNA solutions were annealed using a thermal cycler (Mastercycler nexus X2, Eppendorf) at a cooling rate of 0.1 °C/s; subsequently, the solutions were diluted 2/5 times using the same buffer (100 mM NaCl, 20 mM Tris-HCl (pH 8.0), and 1×SYBR Gold). The DNA solutions and the 100-bp DNA ladder marker solution were mixed with an equal volume of the 2×non-denaturing loading buffer, and 5 *μ*l of the DNA solutions was loaded onto the non-denaturing 8% (w/w) polyacrylamide gels.

### D. UV light irradiation

For UV light irradiation, we used a high-pressure mercury lamp (a handy type UV curing device HLR100T-2, with a 100-W high-pressure mercury lamp HL100G, Sen Special Industrial Co., Ltd.) by adjusting the intensity to 8 mW/cm^2^ at the irradiated site and a xenon lamp with a 313 nm bandpass filter (an ultraviolet irradiation device, MAX-303 Compact Xenon Light Source, Asahi Spectra Co., Ltd.) by adjusting the intensity to 15 mW/cm^2^ at the irradiated site.

### E. Confirmation of the activation of the inactivated linker by UV light irradiation using PAGE

We confirmed the activation of the inactivated linker by UV light irradiation using PAGE [Fig. 3(b)]. In Fig. 3(b), the DNA solution for lane L1–L5 contained 1 *μ*M inactivated linker, 100 mM NaCl, 20 mM Tris-HCl (pH 8.0), and 1×SYBR Gold. The DNA solution for lane L6 contained 1 *μ*M activated linker, 100 mM NaCl, 20 mM Tris-HCl (pH 8.0), and 1×SYBR Gold. The DNA solutions in the PCR tubes (10 *μ*l) were annealed in the thermal cycler at a cooling rate of 0.1 °C/s; then, UV light was irradiated on the PCR tubes containing the DNA solutions with the high-pressure mercury lamp. The sample and the 10-bp DNA ladder marker were mixed an equal volume of 2×denaturing loading buffer and heated at 80 °C for 3 min using the thermal cycler to denature the hybridized DNA. Finally, 5 *μ*l of the DNA solutions were loaded on a denaturing 10% (w/w) polyacrylamide gel with 8 M urea.

### F. Melting curve measurement before/after UV light irradiation

Two DNA solutions (20 *μ*l) consisting of 5 *μ*M inactivated linker, in a buffer of 100 mM NaCl, 20 mM Tris-HCl (pH 8.0), and 1×SYBR Green I, were prepared in PCR tubes. UV light was irradiated on only one of the PCR tubes with the high-pressure mercury lamp for 3 min. The fluorescence intensity of the two samples (UV-light irradiated sample and non-irradiated sample) was measured using the fluorescence intensity detection mode of a real-time PCR system (CFX Connect, Bio-Rad). The fluorescence intensity was measured every 1 °C with decreasing temperature from 90 °C to 25 °C. The averaged fluorescence intensity of three samples was calculated and then the normalized intensity was calculated by dividing the averaged intensity by the intensity at 85 °C and subtracting 1 from the divided value [Fig. 3(c)].

### G. Confirmation of gelation by UV light irradiation using PAGE and microscopy

In Fig. 3(d), DNA solutions with 12.5 *μ*M Y-motif A, 12.5 *μ*M Y-motif B, 12.5 *μ*M inactivated linker, and 12.5 *μ*M activated linker were prepared separately in a buffer containing 100 mM NaCl, 20 mM Tris-HCl (pH 8.0), and 1×SYBR Gold and then annealed at a cooling rate of 0.1 °C/s. Subsequently, by mixing of 1 *μ*l of 12.5 *μ*M Y-motif A, 1 *μ*l of the 12.5 *μ*M Y-motif B, and 3 *μ*l of the 12.5 *μ*M inactivated linker (or 12.5 *μ*M activated linker, or buffer alone), 2.5 *μ*M Y-motif A, 2.5 *μ*M Y-motif B, and 0 or 7.5 *μ*M inactivated/activated linker in 100 mM NaCl, 20 mM Tris-HCl (pH 8.0), and 1×SYBR Gold, was obtained in the PCR tube. For the sample in lane L3, UV light was irradiated on the PCR tube containing the DNA solution for 3 min with the high-pressure mercury lamp. The DNA solutions were mixed with an equal volume of 2×non-denaturing loading buffer; then, 5 *μ*l of the mixed samples was loaded on a non-denaturing 8% (w/w) polyacrylamide gel.

In Fig. 3(e), DNA solutions of 12.5 *μ*M Y-motif A, 12.5 *μ*M Y-motif B, and 12.5 *μ*M inactivated linker, in a buffer of 100 mM NaCl, 20 mM Tris-HCl (pH 8.0), and 1×SYBR Gold were prepared, and they were annealed at the cooling rate of 0.1 °C/s; then, by mixing 1 *μ*l of 12.5 *μ*M Y-motif A, 1 *μ*l of 12.5 *μ*M Y-motif B, and 3 *μ*l of 12.5 *μ*M inactivated linker, 2.5 *μ*M Y-motif A, 2.5 *μ*M Y-motif B, and 7.5 *μ*M inactivated linker in 100 mM NaCl, 20 mM Tris-HCl (pH 8.0), and 1×SYBR Gold, was obtained in the PCR tube. Thereafter UV light was irradiated on the sample for 3 min with the high-pressure mercury lamp.

### H. Fabrication of photomasks

The photomasks were fabricated by photolithography as follows. The photomask design was created using the software Rhinoceros 4.0 (Robert McNeel & Associates). The slide glass (micro cover glass No.5, 0.45–0.60 mm thickness, Matsunami Glass) was cleaned for 90 s with a hydrophilic treatment device (Ion Bomberder, Vacuum Device Co., Ltd.). Then, the glass slide was enclosed in an aluminum container with 100 *μ*l of HMDS. The container was sealed and heated to 90 °C for 1 h in order to improve photoresist adhesion. The photoresist S1818 was spin-coated on the treated glass slide. The spin coating device (Opticoat SpinCoater, Mikasa Co., Ltd.) was used for spin coating at a maximum spin frequency of 3 000 rpm for 30 s. After the resist material was coated on the slide glass, the glass slide with photoresist was pre-baked for 1 min at 115 °C and left to cool to down to room temperature (approximately 25 °C). To expose the photoresist, we used a mask-less pattern generator *μ*PG 101 (laser wavelength 375 nm, Heidelberg Instruments Mikrotechnik GmbH). After exposure, the photoresist was developed using the developer NMD and cleaned with IPA. Thereafter, a metal deposition device (VE2012 TMP vacuum evaporator, Vacuum Device Co., Ltd.) was used for the deposition of chromium and gold, in this order. The remaining photoresist was removed using an acetone bath in an ultrasonic cleaner.

### I. Formation of shape-controlled DNA-motif hydrogels with UV light irradiation

First, the DNA solutions of 100 *μ*M FAM-labeled Y-motif A (100 *μ*M YA-1, 75 *μ*M YA-2, 25 *μ*M Y-2_FAM, and 100 *μ*M YA-3) in a buffer of 200 mM NaCl and 20 mM Tris-HCl (pH 8.0), 100 *μ*M FAM-labeled Y-motif B (100 *μ*M YB-1, 75 *μ*M YB-2, 25 *μ*M Y-2_FAM, and 100 *μ*M YB-3) in the same buffer, and 150 *μ*M inactivated linker in the same buffer were prepared and annealed at a cooling rate of 0.1 °C/s. Next, by mixing 1.5 *μ*l of the 100 *μ*M FAM-labeled Y-motif A, 1.5 *μ*l of the 100 *μ*M FAM-labeled Y-motif B, and 3 *μ*l of the 150 *μ*M inactivated linker, 25 *μ*M FAM-labeled Y-motif A, 25 *μ*M FAM-labeled Y-motif B, and 75 *μ*M inactivated linker dissolved in the buffer of 200 mM NaCl and 20 mM Tris-HCl (pH 8.0), was obtained in the PCR tube.

To make the gelation chamber [Fig. 4(a)], an 18 × 18-mm-sized square glass slide (0.13–0.17 mm thickness, Matsunami Glass) and a 30 × 40-mm-sized square glass slide (0.13–0.17 mm thickness, Matsunami Glass) were used as the upper and lower glass slides, respectively. The glass slides were treated with O_2_ plasma for 3 min using the hydrophilic treatment device. Then, the gelation chamber was constructed by attaching the upper cover glass to the lower cover glass with double-faced adhesive tape [Fig. 4(a)]. In order to prevent the adsorption of DNA to the glass surface of the gelation chamber, BSA was coated on the glass surface. This was done by injecting 5% (w/v) BSA, dissolved in 20 mM Tris-HCl buffer (pH 8.0), into the gelation chamber. Subsequently, the gelation chamber was incubated for 20 min at room temperature together with water in a closed vessel, in order to prevent evaporation of the BSA solution [Fig. S4(a)]; then, the BSA solution was washed away with 10 *μ*l of 20 mM Tris-HCl buffer (pH 8.0), followed by a second washing step, after 10 min.

We injected 6 *μ*l of the DNA solution for the experiments of Figs. 4 and 5 or 10 *μ*l of the DNA solution for the experiments of Fig. 6 and irradiated UV light with the xenon lamp with 313 nm bandpass filter for 10 min. During UV light irradiation, the gelation chamber was placed together with water in a Petri dish covered with a quartz glass plate in order to prevent evaporation of the DNA solution [Fig. S4(b)]; the photomask was placed on the quartz glass plate. After UV light irradiation, the DNA-motif hydrogel was observed.

### J. Microscopic observation

To observe the photomasks and the DNA-motif hydrogels formed in the shapes of “D,” “N,” “A,” and a square, we used a fluorescence microscope (IX71, Olympus), a mercury lamp (USH-1030L, Olympus) as the excitation source, an objective lens PLAPON1.25X (Olympus), a sCMOS camera (Zyla, ANDOR), and a control software IQ3 (ANDOR). Subsequently, the fluorescence of FAM-labeled Y-motifs A and B was observed. To evaluate the resolution, cross-shaped structures were observed using a confocal laser scanning microscope (FV1000, Olympus), a control software FV10ASW4.0 (Olympus), and an objective lens UPLFLN4X (Olympus).

### Ethics approval

No ethics approval is required for this work.

## SUPPLEMENTARY MATERIAL

See the supplementary material for additional experimental data and information.

## AUTHORS CONTRIBUTIONS

Y.K. and Y.S. contributed equally to this work.
